# Imaging antiferromagnetic domain fluctuations and the effect of atomic scale disorder in a doped spin-orbit Mott insulator

**DOI:** 10.1126/sciadv.abi6468

**Published:** 2021-11-10

**Authors:** He Zhao, Zach Porter, Xiang Chen, Stephen D. Wilson, Ziqiang Wang, Ilija Zeljkovic

**Affiliations:** 1 Department of Physics, Boston College, Chestnut Hill, MA 02467, USA.; 2 Materials Department, University of California, Santa Barbara, Santa Barbara, CA 93106, USA.

## Abstract

Correlated oxides can exhibit complex magnetic patterns. Understanding how magnetic domains form in the presence of disorder and their robustness to temperature variations has been of particular interest, but atomic scale insight has been limited. We use spin-polarized scanning tunneling microscopy to image the evolution of spin-resolved modulations originating from antiferromagnetic (AF) ordering in a spin-orbit Mott insulator perovskite iridate Sr_3_Ir_2_O_7_ as a function of chemical composition and temperature. We find that replacing only several percent of lanthanum for strontium leaves behind nanometer-scale AF puddles clustering away from lanthanum substitutions preferentially located in the middle strontium oxide layer. Thermal erasure and reentry into the low-temperature ground state leads to a spatial reorganization of the AF puddles, which nevertheless maintain scale-invariant fractal geometry in each configuration. Our experiments reveal multiple stable AF configurations at low temperature and shed light onto spatial fluctuations of the AF order around atomic scale disorder in electron-doped Sr_3_Ir_2_O_7_.

## Teaser

Spin-polarized scanning tunneling microscopy reveals spatial rearrangement of antiferromagnetic puddles in an iridium oxide.

## INTRODUCTION

Competing energy scales in correlated electron systems often lead to granular nature (*1*–*7*), with substantial spatial variations in charge, spin, and orbital degrees of freedom. Depending on the material and the emergent property, the characteristic length scale of this inhomogeneity can span several orders of magnitude. For instance, charge inhomogeneity ranges from several micrometers in manganites (*2*) and Hg-based cuprates (*3*), to a few nanometers in Bi-based cuprates (*1*) and iridates (*4*, *5*). Spin inhomogeneity in correlated quantum solids can exhibit an even more elaborate texture. A ferromagnet can have microscopic domains, each with a different orientation of the local magnetic moment (*6*), sometimes coexisting with spatially separated nonmagnetic regions (*7*). In the presence of antisymmetric spin interactions, spins can gradually twist and form complex, long wavelength spin textures such as skyrmion lattices (*8*–*10*). While a larger spatial extent of ferromagnetic domains and skyrmion spin texture facilitates imaging by a variety of probes, antiferromagnetic (AF) ordering has been notoriously difficult to visualize. This is, in large part, due to the cancellation of signals from neighboring spins, which limits the use of probes that are unable to achieve subnanometer resolution. Tremendous progress has recently been made in imaging micron size AF domains using scattering techniques (*11*). However, to fully understand the nature of the strongly correlated states and quantum fluctuations, it is highly desirable to visualize the formation of AF clusters with atomic resolution, including their size, shape, and distribution in the presence of atomic defects and across different phase transitions.

Over the past decade, Ruddlesden-Popper iridates (*12*–*15*) have emerged as an intriguing family of two-dimensional (2D) AF spin-orbit Mott insulators, where the AF Mott ground state can be tuned by different parameters, for example, temperature and chemical composition. The experiments thus far revealed a remarkable nanoscale phase separation in iridates, reflected in the granular fabric of both their electronic and magnetic properties (*4*, *5*, *16*). While, in many correlated oxides, the competition between different phases provides a natural tendency toward phase separation (*5*, *17*, *18*), chemical disorder that is often necessary to induce a new phenomenon can also play a substantial role (*5*, *19*, *20*). Our recent spin-polarized scanning tunneling microscopy (SP-STM) experiments revealed the emergence of AF puddles near insulator-to-metal transition in the Ruddlesden-Popper iridate Sr_2_IrO_4_ (*16*). However, many questions remain regarding the formation of AF clusters in this family of oxides. For example, how sensitive is the domain size and shape to the inevitable presence of chemical disorder? How repeatable is the AF pattern configuration after it is thermally wiped out by increasing the temperature and then cooled back down? In this work, we explore these questions in the bilayer iridate Sr_3_Ir_2_O_7_ (Ir-327). Ir-327 is less insulating compared to its single-layer counterpart Sr_2_IrO_4,_ which facilities tunneling measurements across a wider doping range down to the near “parent” state. Using SP-STM, we find that multiple AF configurations can nucleate at low temperature and reveal the effects of different types of disorder on the local distribution of AF ordering.

## RESULTS

**Fig. 1.**
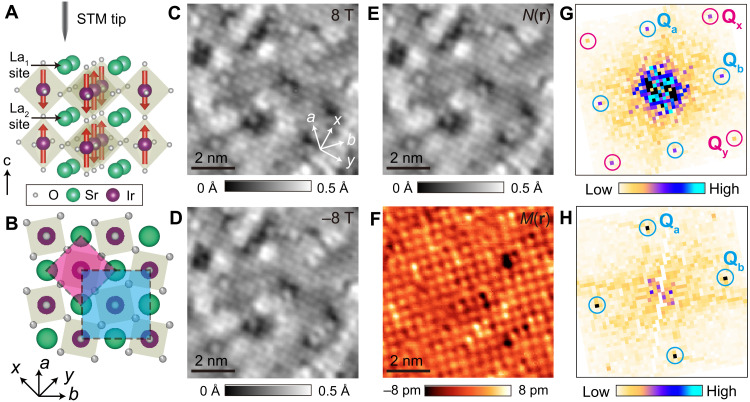
Schematic of the crystal structure and the magnetic structure of Ir-327 and SP-STM imaging. (**A**) 3D crystal structure and (**B**) *ab*-plane atomic structure of Ir-327. Red arrows in (A) denote the direction of Ir spin moment. Two unit cells are outlined by squares in (B): the Sr lattice unit cell (light pink) and the magnetic unit cell (light blue). (**C** and **D**) STM topographs *T*(**r**, **B**) of near-parent Ir-327 acquired using a spin-polarized tip, in magnetic field of **B** = +8 T (C) and **B** = −8 T (D) applied perpendicular to the sample surface [+(−) sign denotes the field parallel (antiparrallel) to **z**]. (**E**) *N*(**r**) map, defined as the arithmetic average of STM topographs in (C) and (D). (**F**) Spin-resolved magnetic contrast *M*(**r**) map, obtained by the subtraction of STM topographs in (C) and (D), box car averaged with a 1-pixel radius. (**G** and **H**) Fourier transform of *N*(**r**) and *M*(**r**) maps shown in (E) and (F), respectively. **Q**_**x**_ and **Q**_**y**_ represent Sr lattice atomic Bragg peaks, while **Q**_**a**_ and **Q**_**b**_ denote the Fourier peaks related to spin-resolved AF modulations. STM setup condition: *V*_sample_ = 1 V, *I*_set_ = 30 pA, **B** = ±8 T.

**Fig. 2.**
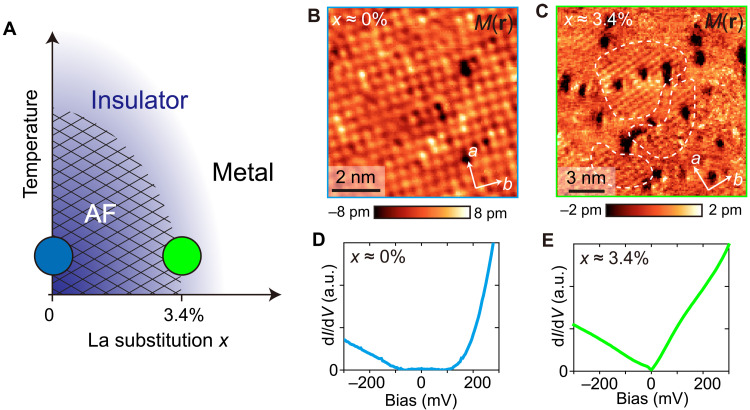
Evolution of spin-resolved modulations and differential conductance as a function of composition in (La*_x_*Sr_1−*x*_)_3_Ir_2_O_7_. (**A**) Schematic phase diagram of (La*_x_*Sr_1−*x*_)_3_Ir_2_O_7_ as a function of La concentration *x*. The squared mesh roughly outlines the AF order phase detected by magnetization measurements and scattering experiments (*23*, *24*). The purple (white) background represent the insulator (metal) phase. Two different compositions measured in this work are near parent *x* ~ 0% (blue circle) and *x* ~ 3.4% (green circle). (**B** and **C**) Spin-resolved *M*(**r**) maps (box car averaged by 1-pixel radius), and (**D** and **E**) associated spatially averaged d*I/*d*V*(**r**,V) spectra for (B and D) *x* ~ 0% and (C and E) *x* ~ 3.4% sample. STM setup condition: (B) *V*_sample_ = 1 V, *I*_set_ = 30 pA, ±8 T; (C) *V*_sample_ = 600 mV, *I*_set_ = 100 pA, ±4 T; (D) *V*_sample_ = 400 mV, *I*_set_ = 100 pA, *V*_exc_ = 4 mV, 0 T; and (E) *V*_sample_ = 300 mV, *I*_set_ = 300 pA, *V*_exc_ = 6 mV, 0 T. a.u., arbitrary units.

Undoped parent Ir-327 exhibits collinear AF ordering below Neel temperature (*T*_N_) ~ 285 K, with local magnetic moment of the Ir atoms pointing out-of-plane (Fig. 1A) (*21*, *22*). On the basis of the combination of magnetization and neutron scattering measurements, the long-range AF order can be suppressed by temperature or by chemical substitutions (Fig. 2A) (*23*, *24*). To access local spin information related to the AF order in this system, we apply SP-STM imaging (see Materials and Methods and fig. S1) (*25*). We acquire a pair of STM topographs over an identical area of the sample in magnetic field applied parallel and antiparallel to the **c** axis (Fig. 1, C and D). The reversal of the magnetic field direction serves to “flip” the spin polarization of the STM tip, without notably affecting the magnetic moment orientation in the sample. By subtracting the two topographs, we can extract the spin-resolved magnetic contrast map *M*(**r**), which shows prominent modulations (Fig. 1F). The wave vectors of these modulations, **Q**_**a**_ = (0.5, −0.5) and **Q**_**b**_ = (0.5, 0.5) (we hereafter define reciprocal lattice vector 2π/*a*_0_ = 1), are identical to those attributed to long-range AF order by neutron scattering experiments (*23*, *24*).

Using this procedure, we investigate the evolution of spin-resolved modulations in Ir-327 as a function of La substituting for Sr (Fig. 2, A to C) (*24*, *26*), while, at the same time, tracking the electronic properties from differential conductance d*I*/d*V* spectra (Fig. 2, D and E). While, in the near parent state, we find that the spin-resolved modulations appear spatially uniform (Fig. 2B), in *x* ~ 3.4% La-substituted sample, we reveal a spatial inhomogeneity in the *M*(**r**) maps, with distinct regions where modulations are completely absent (Fig. 2C). This spatial inhomogeneity is also reflected in the larger width of the Fourier space peaks at **Q**_**a**_ and **Q**_**b**_ (fig. S1). The inhomogeneous disappearance of the AF order with La substitution in the bilayer iridate Ir-327 is consistent with our observations in La-substituted Sr_2_IrO_4_ (*16*), suggesting that the granular nature of the AF order near the transition is not limited to a single iridate. Moreover, out-of-plane spins in Ir-327 allow a complete mapping of all AF domains, in contrast to Sr_2_IrO_4_ where in-plane spin orientation may lead to some domains being undetectable by a SP-STM tip (see Materials and Methods).

**Fig. 3.**
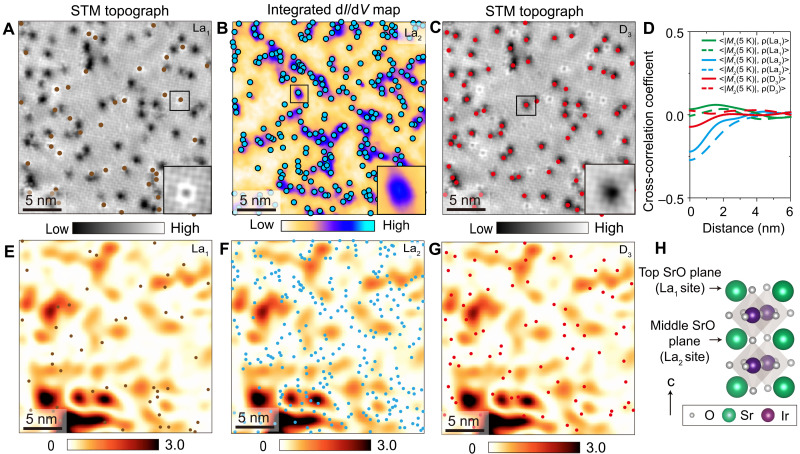
Cross-correlation between spin-resolved magnetic contrast map *M*(r) and defect distribution in *x* ~ 3.4% Ir-327 sample. (**A** and **C**) STM topograph and (**B**) integrated d*I*/d*V* (**r**, *V*) map from −36 mV to 66 mV acquired over the same region of the sample. The positions of three different types of dopants in (A) to (C) are denoted by circles of different color: La_1_ (substitution for Sr in the top SrO plane) [brown dots in (A) and (E)], La_2_ (substitution for Sr in the middle SrO plane) [light blue dots in (B) and (F)], and another unidentified defect labeled D_3_ [red dots in (C) and (G)]. The insets in (A) to (C) show zoom-ins on each type of defect. (**D**) Radially averaged cross-correlation between *M*(**r**) amplitude maps at 5 K [initial cooldown |*M*_1_(**r**, 5 K)| and after thermal cycling |*M*_2_(**r**, 5 K)|] and dopant density maps (fig. S4). (**E** to **G**) *M*(**r**) amplitude map |*M*_1_(**r**, 5 K)| with positions of different defects superimposed: La_1_ (E), La_2_ (F), and D_3_ (G). (**H**) A 3D crystal structure of one unit cell of Ir-327. The two inequivalent La substitution sites are marked by black arrows: La_1_ (substitution for Sr in the top or the bottom SrO plane) and La_2_ (substitution for Sr in the middle SrO plane). STM setup conditions: (A and C) *V*_sample_ = 600 mV, *I*_set_ = 100 pA, 4 T; and (B) *V*_sample_ = 300 mV, *I*_set_ = 200 pA, *V*_exc_ = 6 mV, 0 T.

Next, we set to explore whether and how chemical disorder affects the AF texture observed after introducing a dilute concentration of La substitutions. We focus on the three types of defects that we most commonly observe in this system: La substitution for Sr in the topmost SrO plane seen as bright squares in STM topographs (La_1_), La substitution for Sr in the second SrO layer seen as bright features in d*I*/d*V*(**r**, *V*) maps at low energy (La_2_) (*27*), and dark features in STM topographs (D_3_) (Figs. 1A and 3, A to C and H, and figs. S3 and S4). To visualize how the AF order arranges itself relative to local disorder, we superimpose the positions of each type of defect on top of the local AF amplitude map |*M*(**r**)| (Fig. 3, E to G). Visually, the AF order appears to be the strongest away from La_2_ substitutions, while La_1_ and D_3_ defects are nearly randomly distributed with respect to the AF order. This conclusion is supported by the cross-correlation of the |*M*(**r**)| maps and dopant density maps ρ(***r***) (Fig. 3D and fig. S4), which shows a cross-correlation coefficient α ~ −0.3 for La_2_, while α coefficients for La_1_ and D_3_ defects are substantially weaker. Bulk measurements of La-doped Ir-327 found that long-range AF order is gradually suppressed with the increase in La concentration (*24*). Our SP-STM experiments are consistent with this global picture and further reveal that the inhomogeneous AF order near the transition point has a tendency to weaken in proximity to a particular type of La disorder, located in the middle SrO plane.

**Fig. 4.**
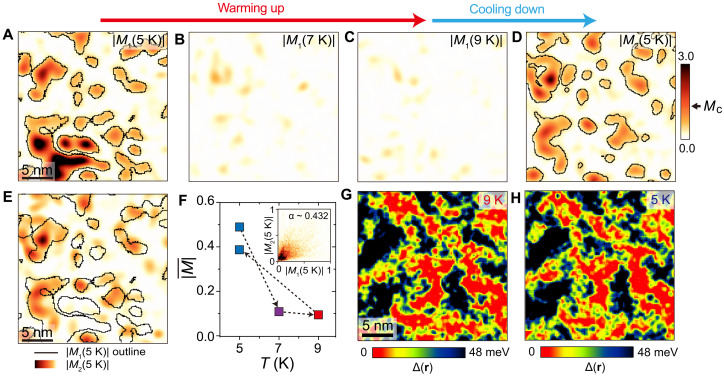
Evolution of magnetic and electronic properties of *x* ~ 3.4% La-substituted Ir-327 along a thermal loop. (**A** to **D**) AF amplitude maps |*M*(**r**, *T*)| as a function of temperature, all acquired over the same region under identical conditions. Magnetic textures outlined in (A) and (D) have been extracted using the same amplitude threshold value *M*_c_. (**E**) AF amplitude map |*M*_2_(**r**, 5 K)| overlapped with a magnetic texture outline of |*M*_1_(**r**, 5 K)| map from (A). (**F**) Plot of the average value of |*M*(**r**, *T*)| in (A) to (D). Inset in (F) is a 2D intensity cross-correlation histogram of |*M*_1_(**r**, 5 K)| and |*M*_2_(**r**, 5 K)|. (**G** and **H**) Maps of the spectral gap Δ(**r**) [section S3 and (*16*, *34*, *35*)] at 9 and 5 K, respectively, both obtained over the same area as |*M*(**r**, *T*)| maps. STM setup condition: *M*(**r**) maps: *V*_sample_ = 600 mV, *I*_set_ = 100 pA, ±4 T, 5 K (A), 7 K (B), 9 K (C), and 5 K (D); Δ(**r**) maps: *V*_sample_ = 300 mV, *I*_set_ = 200 pA, *V*_exc_ = 6 mV, 0 T, 9 K (G) and 5 K (H).

In general, strong disorder pinning could suggest that a spatial pattern associated with the AF order will be repeatable after cycling through the magnetic transition and back. To investigate this, we image the evolution of spin-resolved AF modulations as a function of temperature, tracking the same area of the sample (Fig. 4, A to D). We find that the average |*M*(**r**)| signal quickly subsides by ~9 K (Fig. 4F). This provides further evidence that our sample is near the transition point at this La composition. We also note that the spectral gapmaps at 4.5 and 9 K are nearly identical (Fig. 4, G and H), thus confirming a lack of direct dependence of the most prominent spectral gap to the local AF order (*16*). After increasing the temperature to ~10 K, we cool the sample back down to ~4.5 K and repeat the measurement. Although the cross-correlation between the AF amplitude maps before (|*M*_1_(5 K)|) and after thermal cycling (|*M*_2_(5 K)|) is relatively high (Fig. 4F, inset), we observe distinct changes in the patterns (Fig. 4E), indicating a change in the low-temperature AF configuration. In both cases, however, the AF patterns form away from La_2_ substitutions, while other defects play a minor role (Fig. 3, E to G).

**Fig. 5.**
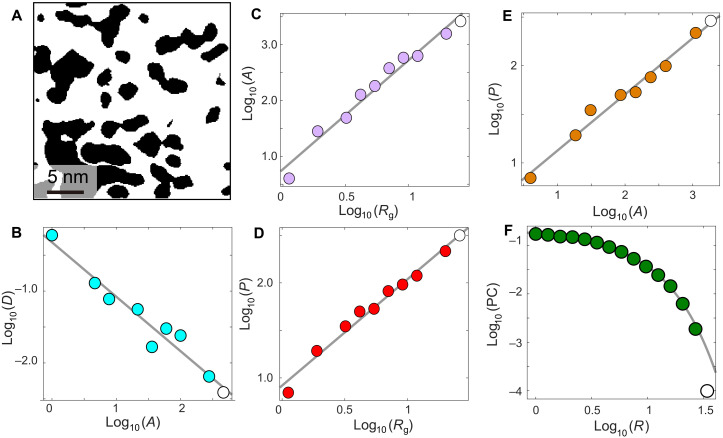
Scale-invariant magnetic texture at 5 K. (**A**) Binarized AF domains obtained from |*M*_1_(**r**, 5 K)| based on an amplitude threshold *M*_c_. (**B**) Logarithmically binned domain area distribution *D*(*A*), following a scale-free power law distribution [*D*(*A*) ~ *A*^−τ^] with the critical exponent τ = 0.76 ± 0.18. (**C** and **D**) Area (*A*) and perimeter (*P*) versus gyration radius (*R*_g_) plotted using logarithmic binning. Solid lines are power law fits of *P* ~ *R*_g_^dh*^ and *A* ~ *R*_g_^dv*^, with critical exponents dh^*^ = 1.12 ± 0.04 and dv^*^ = 1.95 ± 0.18. (**E**) Perimeter (*P*) versus area (*A*) plot, reflecting the effective fractal dimension ratio dh^*^/dv^*^. The solid line is the power law fit of *P* ~ A^dh*/dv*^ with dh^*^/dv^*^ ~ 3/5. (**F**) Pair connectivity (PC) function versus distance (*r*) plot using logarithmic binning. The solid line is fit to a power law function with an exponential cutoff *g*_conn_ ~ *r*^−η^**·***e*^−*x/*ξ^, where ξ is the correlation length and η = 0.05 ± 0.02 is the exponent for the connectivity function. Values of *P*, *r*, and *R*_g_ are in units of pixels and *A* is in units of area occupied by a single pixel. The single pixel size in the raw STM topograph used to obtain image in (A) is 1.2 Å by 1.2 Å.

The ability to visualize the AF clusters in real space allows us to investigate their size, shape, and distribution in more detail. We apply 2D cluster analysis theory, a powerful statistical analysis tool that can be used to probe the strength of electronic correlations and near-critical behavior from the geometric metrics of the clusters (*11*, *28*–*30*). We first binarize the AF amplitude map |*M*_1_(5 K)| based on an intensity cutoff (Fig. 5A and section S2). Then, using logarithmic binning, a standard technique for power law distribution analysis (*31*), we plot the relationship between several geometrical descriptors of the clusters: area *A*, perimeter *P*, gyration radius *R*_g_ [defined as $2R_{g}^{2}=\sum_{i,j}\frac{{|r_{i}-r_{j}|}^{2}}{s^{2}}$, where *r*_*i*(*j*)_ is the position of the *i*(*j*)th site in the cluster (*32*) and *s* is the size of the cluster], number of clusters of given area *D*(*A*), and pair connectivity PC(*r*) (defined as the probability that two sites separated by a distance *r* belong to the same connected finite cluster). In the analysis, we exclude the clusters touching the boundaries, as we do not have a complete set of geometric metrics for these. We find that the domain area distribution histogram *D*(*A*) versus *A* shows a linear dependence on a logarithmic scale spanning over 2.5 decades. By fitting *D*(*A*) = *A*^−τ^, we determine the critical exponent τ = 0.76 ± 0.18 (Fig. 5B). Next, we study the relationship between the area *A* (perimeter *P*) and the gyration radius *R*_g_ across all domains, as shown in Fig. 5C (Fig. 5D). We find that both *A* and *P* scale as a power of *R*_g_ (*P* = *R*_g_^dh*^ and *A* = *R*_g_^dv*^) for exponents dv* (dh*) often referred to as effective volume (hull) dimensions (*32*). From the fitting analysis, we determine these to be dv* = 1.95 ± 0.18 and dh* = 1.12 ± 0.04. To put these values in perspective, we note that coefficients for an uncorrelated percolation should be dv* ≈ 1.90 and dh* = 1.75 (*32*). The difference between these coefficients and those extracted from our data suggests that the formation of AF patterns in our sample may be influenced by electronic correlations in the presence of disorder (section S2). To further support the validity of our cluster analysis, we point to the following. First, the exponent ratio dh*/dv* determined directly from the slope in Fig. 5E (3/5 ± 0.04) is nearly identical to the ratio of dh* and dv* calculated individually from Fig. 5 (C and D). Second, we note that the hyperscaling relation, *d −* 2 *+* η *=* 2 (d − dv*), expected to hold near the transition point within this model, is satisfied (section S2). Here, *d* is the dimensionality of the system taken to be 2 given the quasi-2D nature of Ir-327, and η = 0.05 ± 0.02 is the coefficient obtained from fitting pair connectivity function PC(*r*) *~ r*^−η^**·***e*^−r/ξ^ (Fig. 5F).

## DISCUSSION

Our experiments provide a new insight into the inhomogeneous distribution of spin degrees of freedom relative to chemical disorder, complementary to the reports of nanoscale separation in the charge degrees of freedom in the same family of materials (*4*, *23*, *27*). We find that chemical disorder can put some constraints on the morphology of the AF texture. At the same time, thermal cycling experiments reveal substantial spatial fluctuations and rearrangement of the AF puddles around this disorder. While the AF distribution maps at different temperatures appear markedly different (Fig. 4, C and D), spectral gapmaps look virtually identical (Fig. 4, G and H), thus confirming that short-range AF order is not the cause behind the spatially inhomogeneous gap closing (*16*). While our work points toward La_2_ substitutions in the middle SrO layer having the strongest correlation with the regions where the AF order is absent, it remains to be seen what is the physical effect driving this behavior. The site-dependent effect of La substitutions on the local distribution of the AF order is particularly puzzling and warrants further experimental and theoretical work.

Revealing scale invariance of the AF order in real space near the doping induced transition is an important step toward understanding the correlation physics in this spin orbit–coupled Mott system. The statistical analysis of the cluster geometric metrics suggests that the disappearance of the AF order exhibits signatures of a continuous phase transition near criticality. However, we caution that the comparison of the exponents to the oversimplified model of uncorrelated classical percolation provides only a simple benchmark for the possible emerging continuous transition. The deviation of the exponents from the percolation model also points toward the role of electronic correlations, which can influence the magnetic correlation length and, as such, the size of the clusters. Note that even for a percolative transition, electronic correlations can still play a role since they may control the low-energy excitations at the boundary of the clusters where the electrons can quantum tunnel at low temperature to other clusters or to the nonmagnetic region. While, in this work, we reveal spatial variations of the AF order parameter, future experiments could attempt to detect AF fluctuations in time domain by measuring step-like jumps in tunneling current in constant height STM mode, in analogy to sudden changes in the optical response in 2D magnets (*33*). By exploring the evolution of the coherence length with temperature in the statistical analysis of fractal patterns, future experiments focusing on large sample areas to gain higher statistics could shed more light on the underlying criticality in this family of iridates.

## MATERIALS AND METHODS

### Sample synthesis

Single crystals of (Sr_1−*x*_La*_y_*)_3_Ir_2_O_7_ were synthesized via a flux growth technique. High-purity powders of SrCO_3_, IrO_2_, and La_2_O_3_ (Alfa Aesar) were dried, and stoichiometric amounts were measured out, in a 15:1 molar ratio between SrCl_2_ flux and the target composition. Powders were loaded into a platinum crucible and further contained inside alumina crucibles to limit volatility. Mixtures were heated to 1300°C, slowly cooled to 850°C at a rate of 3.5°C hour^−1^, and then furnace-cooled to room temperature. The resulting boule was etched with deionized water, revealing black plate-like crystals with typical dimensions of ~1 mm by 1 mm by 0.1 mm. More detailed chemical, structural, and electronic characterization can be found in (*24*).

### STM characterization

(Sr_1−*x*_La*_x_*)_3_Ir_2_O_7_ samples were cleaved in ultrahigh vacuum at ~80 K and immediately inserted into the STM head. All STM data were acquired using a customized Unisoku USM1300 system at the base temperature of ~5 K, unless otherwise noted. A typical STM topograph of reveals a square lattice of Sr atoms in the topmost SrO plane, with the lattice constant *a*_0_ ~ 0.39 Å (Fig. 1, C and D). To produce the spin-resolved magnetic contrast *M*(**r**) maps, the Lawler-Fujita drift-correction algorithm (*34*) was applied to eliminate the inevitable effects of piezo hysteresis and thermal drift and align the topographic images acquired at two different fields using various defects as position markers. Spectroscopic measurements were taken using a standard lock-in technique at 915-Hz frequency and varying bias excitation as detailed in the figure captions.

Spin-polarized tips were electrochemically etched from a Cr wire in 2 M NaOH solution, similar to the process described in our previous work (*16*). The tip was trained to be ferromagnetic on the surface of ultrahigh vacuum–cleaved single crystals of antiferromagnet Fe_1+*y*_Te by fast scanning and bias pulsing to produce the desired sharpness and the spin-resolved contrast. An advantage of a spin-polarized tip prepared in this method is low “stray” magnetic field because the bulk of the tip remains AF. Last, we note that, in the single-layer iridate Sr_2_IrO_4_, spins are oriented in-plane, which can lead to AF domains with spins rotated by 90° in-plane (*16*). This can, in principle, lead to some AF domains that are undetectable by an STM tip, if tip polarization is exactly orthogonal to the spin orientation of the sample [see more details in supplementary information of (*16*)]. This is not the case in Ir-327 because spins point out-of-plane and no orthogonally oriented domains should exist.

## Supplementary Materials

### This PDF file includes:

Sections S1 to S3 Table S1 Figs. S1 to S9 References
