# Supplementary material for: Imaging antiferromagnetic domain fluctuations and the effect of atomic scale disorder in a doped spin-orbit Mott insulator
Source: Sci Adv. 2021 Nov 10;7(46):eabi6468. doi: 10.1126/sciadv.abi6468 (PMC8580306; doi:10.1126/sciadv.abi6468)
Supplement: Supplementary file 1 — Sections S1 to S3 Table S1 Figs. S1 to S9 References [file sciadv.abi6468_sm.pdf]

Science Advances
AAAS

Supplementary Materials for

Imaging antiferromagnetic domain fluctuations and the effect of atomic scale disorder in a doped spin-orbit Mott insulator

He Zhao, Zach Porter, Xiang Chen, Stephen D. Wilson, Ziqiang Wang, Ilija Zeljkovic*

*Corresponding author. Email: ilija.zeljkovic@bc.edu

Published 10 November 2021, *Sci. Adv.* **7**, eabi6468 (2021)
DOI: 10.1126/sciadv.abi6468

This PDF file includes:

Sections S1 to S3
Table S1
Figs. S1 to S9
References

1 Determination of La concentration from STM data

There are two different lattice sites where La can substitute for Sr in $Sr_3Ir_2O_7$ (Ir-327): one in the outermost SrO layer (La_1) and another in the middle SrO plane (La_2)(Figure S3(a)) [27]. Substitutions at La_1 sites (topmost SrO plane) are captured in STM topogaphs (green circles in Figure S3 and Figure S4), while the substitutions at La_2 sites can be seen in differential conductance maps (white dots in Figure S3 and Figure S4). Based on symmetry, the top and the bottom SrO layers should be equivalent, so even though we cannot image the La dopants directly in the bottom SrO layer, we assume their density is the same as those in the top layer. This leads to the following equation for the composition:

$$x = (1/3 \times (\# \text{ of La}_2) + 2/3 \times (\# \text{ of La}_1)) \; / \; ((\text{area of FOV})/\text{a}_0{}^2),$$

where FOV and a_0 are in unit of nm^2 and nm, respectively. Using it, we obtain La concentrations to be $x \sim 0.031$ for Region I ($\sim$ 25 nm) and $x \sim 0.035$ for Region II ($\sim$ 22 nm), which agree well with the bulk composition as determined from energy dispersive X-ray spectroscopy (EDS) ($x \sim 0.034$).

Visually, we find that the inhomogeneous AF modulation texture is potentially correlated with substitutions at La_2 sites − AF modulation domains form away from high La_2 dopants regions (Figure S4). This conclusion is supported by calculating the cross-correlation between the magnetic contrast $M(\mathbf{r})$ amplitude maps (both $|M_1(\mathbf{r}, 5\text{ K})|$and $|M_2(\mathbf{r}, 5\text{ K})|$), and local defect density maps, where we obtained a high coefficient $\alpha \sim$ -0.3 for La_2 dopants (Figure S4j).

2 Discussion of the cluster analysis

Statistical analysis of the characteristics of the AF domain geometry in $x \sim 0.034$ La-substituted Ir-327 sample requires us to first binarize the modulation amplitude maps $|M(\mathbf{r})|$ based on some threshold (M_c). This value to some extent determines the number, size and shape of the created clusters in the binarized $|M(\mathbf{r})|$ maps, but is in principle somewhat arbitrary. An effective percolation analysis should be robust to such uncertainty. More importantly, the selection of the threshold value should be able to filter out most of the noise signal. To show that our main conclusion −

the AF modulation puddles maintain scale-invariant fractal geometry − is reliable, we particularly focus on determining the threshold value M_c, as shown in Figure S5, so that 95% of the noise from the same $M(\mathbf{r})$ map can be filtered out but over 20%pixels remain in the filtered magnetic amplitude $|M(\mathbf{r})|$ map. Figure S5 plots the corresponding domain area distribution $D(A)$, cluster perimeter as a function of gyration radius $P(R_g)$, cluster area $\mathrm{A}(\mathrm{R}_g)$, pair connectivity $PC(R)$ and $P(A)$ functions (Ref. 32) for both before and after thermal cycling. We also summarize the fitted scaling exponents in Table S1. The majority of the exponents are inconsistent with the uncorrelated percolation model.

In the case of the clean 2D Ising model (C-2D) for a correlated system, as discussed in Ref. 35, the critical exponents derived from the self-similarity of the geometric clusters satisfy the hyperscaling relation $d - 2 + \eta = 2(d - dv^*)$, with fractal dimensions dv^* (and dh^*). In this sense, we can corroborate the validity of our percolation model analysis on the AF texture by inserting the SP-STM data-extracted critical exponents into the scaling relation above. For example, inserting the fitted exponents from Figure S5 yields $2 - 2 + (0.02 \pm 0.01)$ (left side) and $2 \times (2 - (1.95 \pm 0.18))$ (right side); therefore the exponents are self-consistent. We note that the code used here to determine critical exponents was used on data from Ref. 28, which produced values of critical exponents that are nearly identical to those reported in that reference. We note that modulation amplitude maps $|M(\mathbf{r})|$ are obtained by encompassing the AF peaks in Fourier space with a circle of radius 0.117/Å. The same radius was used to obtain AF amplitude maps at all temperatures. As it can be seen in Figure S8, the algorithm is able to pick out the regions with strong AF modulations sufficiently well.

3 Determination of dI/dV spectral gap and comparison to previous work

The dI/dV spectral gap at every pixel in the field-of-view is generated by the algorithm used in our former work (Ref. 16), as well as Refs. 36, 37. Linear regression is fitted to both the left and the right shoulder of a $log(d\mathrm{I}/d\mathrm{V})$ curve; the intercept between the two linear fits and the "baseline" conductance marks the approximate gap edges. To demonstrate that our algorithm works sufficiently well in extracting the variation in the local gap size, we plot the average dI/dVspectra in 5 different bins based on the gapmap (Figure S2). We can see that binned spectra display a systematic evolution, as expected based on the average gap within each bin.

Comparison of differential conductance spectra between different experiments is shown in Figure S6. We note that our La doped sample at 3.4% is somewhat more metallic than that in previous work (Ref. 27) at $\sim$ 3.2% (Figure S6a). The overall V spec-tral shape in our work for 3.4% doping is comparable to spectra in previous work at slightly higher composition 4%-4.8% (Figure S6b,c,d).

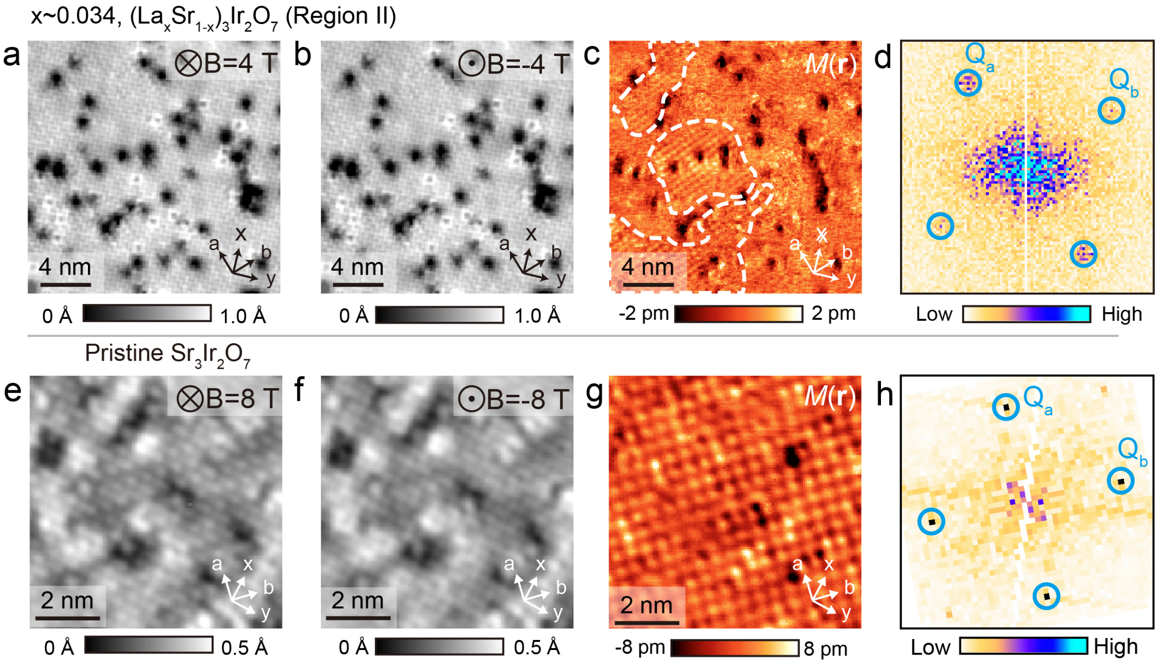

Figure S1. **Spin-polarized imaging of different compositions.** (a,b,e,f) STM topographs of samples obtained with the opposite direction of magnetic field using spin-polarized STM tips. (c,g) $M(\mathbf{r})$ maps, obtained by subtracting STM topographs at two different fields, and (d,h) the corresponding Fourier transform (FT) of the $M(\mathbf{r})$ maps. Spin-resolved modulations appear as Q_a and Q_b (circled in blue color) in the FT images. Dashed lines in the $M(\mathbf{r})$ map in the top row roughly outline the antiferromagnetic domains. STM setup conditions: $x \sim 0.034$: $V_{\text{sample}} = 600$ mV, $I_{\text{set}} = 100$ pA, $\pm$ 4 T; Near pristine: $V_{\text{sample}} = 1$ V, $I_{\text{set}} = 30$ pA, $\pm$ 8 T.

		τ	dh^*	dv^*	η
$\lvert M_1(\mathbf{r}, 5\text{ K})\rvert$	M_c	0.76 ± 0.18	1.12 ± 0.04	1.95 ± 0.18	0.05 ± 0.02
$\lvert M_2(\mathbf{r}, 5\text{ K})\rvert$	M_c	0.88 ± 0.47	1.2 ± 0.02	2.0 ± 0.07	0.02 ± 0.01
	2D uncorrelated percolation model	187/91=2.05	7/4=1.75	91/48=1.90	5/24=0.208

Table S1: Extracted critical exponents for the $|M(5\text{ K})|$ maps from Figure S5 based on the threshold value determined.

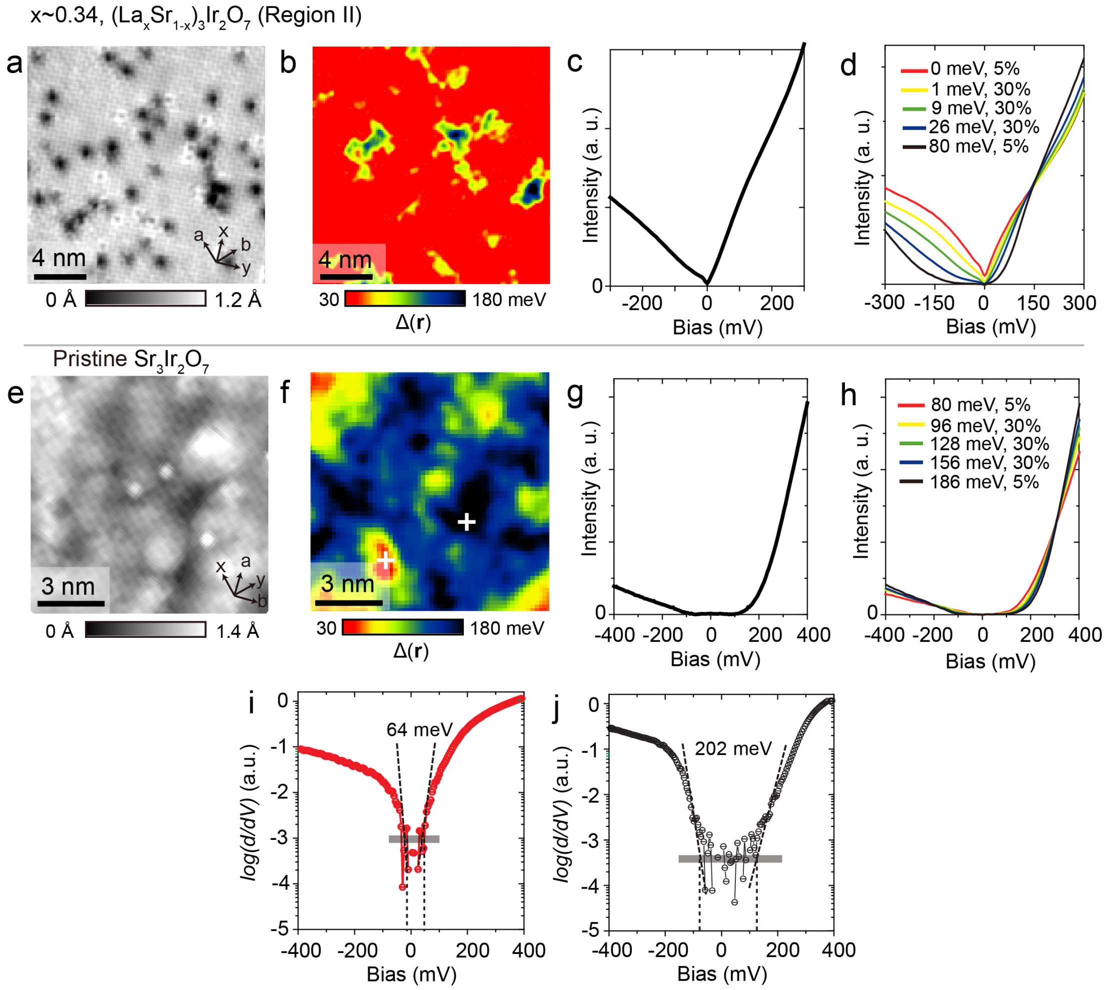

Figure S2. **Spectral gapmaps at different compositions.** (a-d): STM topograph, gapmap, and associated spatially averaged and binned dI/dV spectra obtained over identical regions for $x \sim 0.034$ La-substituted Ir-327, respectively. (e-h): STM topograph, gapmap, and associated spatially averaged and binned dI/dV spectra obtained over identical regions for near-parent Ir-327, respectively. (i,j): Two representative $log(d\mathrm{I}/d\mathrm{V})$ spectra denoted by the two crosses in the gapmap of near-parent Ir-327, associated with small (i) and large (j) gap size. STM setup condition: $x \sim 0.034$: Topograph: $V_{\mathrm{sample}} = 600$ mV, $I_{\mathrm{set}} = 100$ pA, 0 T; Gapmap and spectra: $V_{\mathrm{sample}} = 300$ mV, $I_{\mathrm{set}} = 300$ pA, $V_{\mathrm{exc}} = 6$ mV (zero-to-peak), 0 T; (Near-parent) Topograph: $V_{\mathrm{sample}} = 400$ mV, $I_{\mathrm{set}} = 80$ pA, 0 T; Gapmap and spectra: $V_{\mathrm{sample}} = 400$ mV, $I_{\mathrm{set}} = 100$ pA, $V_{\mathrm{exc}} = 4$ mV, 0 T.

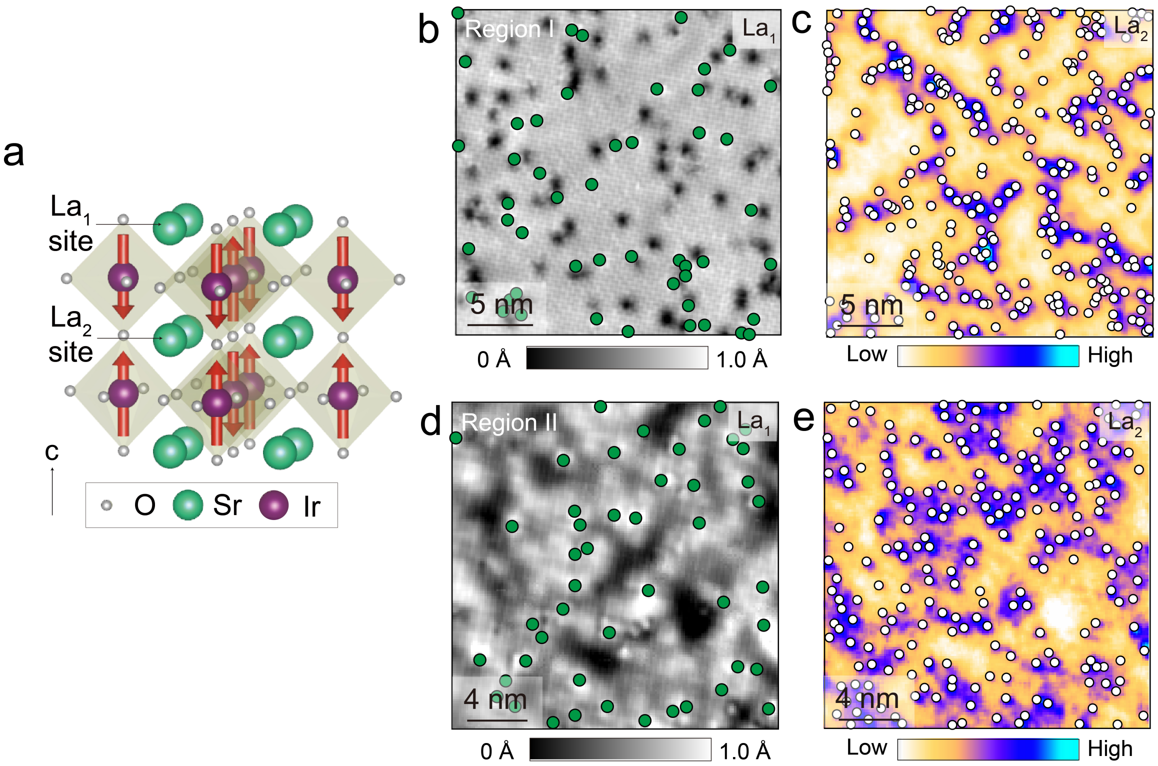

Figure S3. **Two types of La dopants measured in Ir-327**. (a) A 3D crystal structure of $Sr_3Ir_2O_7$. (b,d) STM topographs and (c,e) corresponding integrated differential conductance $dI/dV(\mathbf{r}, V)$ maps from -36 mV to 66 mV over two different regions (labeled I and II) that are approximately micrometer distance away from one another. La substitutions for Sr sites in the top SrO plane (La_1) appear as bright squares in the STM topograph, and are denoted by green dots in (b,d); La substitutions for Sr sites in the middle SrO plane (La_2) are denoted by white dots in (c,e). Counting the number of each substitution type yields 56 La_1 and 279 La_2 in Region I, and 49 La_1 and 211 La_2 in Region II. For visual purposes, integrated conductance images have been box-car-averaged with 1 pixel radius (1 pixel equals 0.1 nm). STM setup condition: (b,c) V_{sample}= 300 mV, I_{set} = 200 pA, V_{exc} = 6 mV, 0 T; (d,e) V_{sample}= -300 mV, I_{set} = 60 pA, V_{exc} = 6 mV, 0 T.

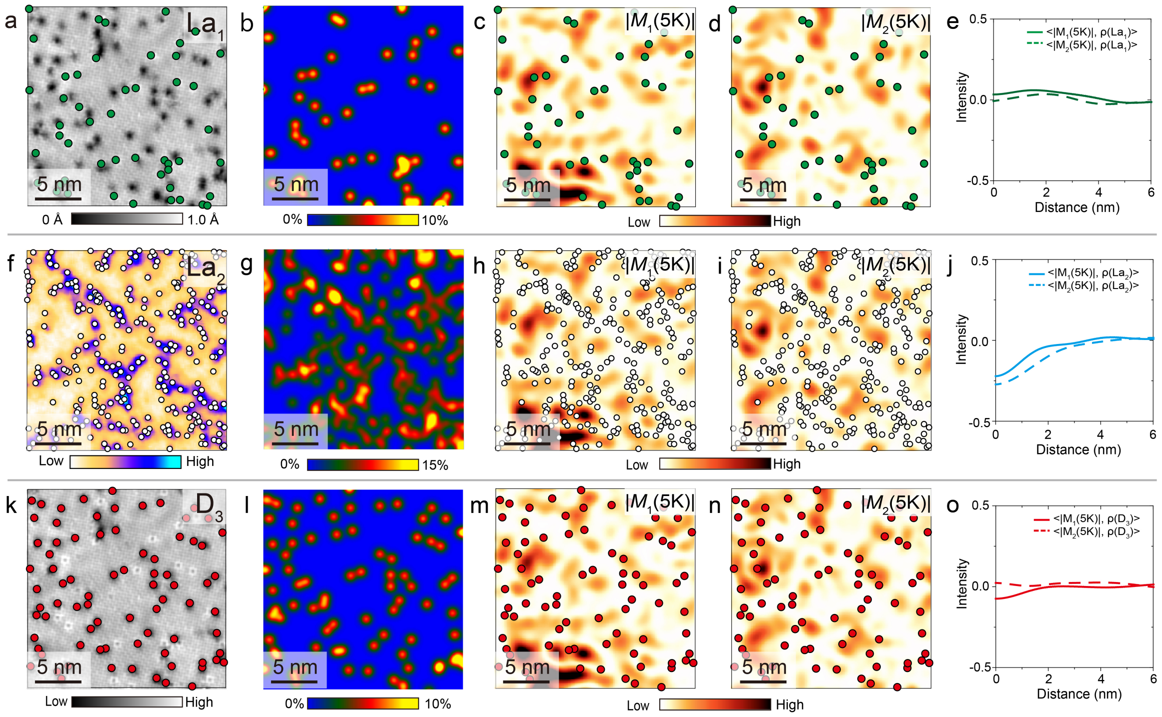

Figure S4. **Cross-correlation between defect density maps and spin-resolved magnetic contrast $M(\mathbf{r})$ map in the $\boldsymbol{x} \sim 0.034$ La-substituted Ir-327 sample**. (a,k) STM topograph and (f) integrated $\mathrm{d}I/\mathrm{d}V(\mathbf{r}, V)$ map from -36 mV to 66 mV acquired over Region I. (b,g,l) La_1, La_2 and D_3(unidentified defects) dopants density map, respectively, with each dopant being represented by a normalized Gaussian with $\sigma \sim 0.5$ nm. Spin-resolved magnetic contrast amplitude maps: (c,h,m) before ($|M_1(5\text{ K})|$) and (d,i,n) after ($|M_2(5\text{ K})|$) thermal cycling. (e,j,o) Plots of cross-correlation between La_1, La_2 and D_3 dopants density map and the $|M(5\text{ K})|$ maps. STM setup condition: Topograph and integrated conductance maps: $V_{\text{sample}}=$ 300 mV, $I_{\text{set}} = 200$ pA, $V_{\text{exc}} = 6$ mV, 0 T; $|M(\text{T})|$ maps: $V_{\text{sample}} = 600$ mV, $I_{\text{set}} = 100$ pA, $\pm$ 4 T.

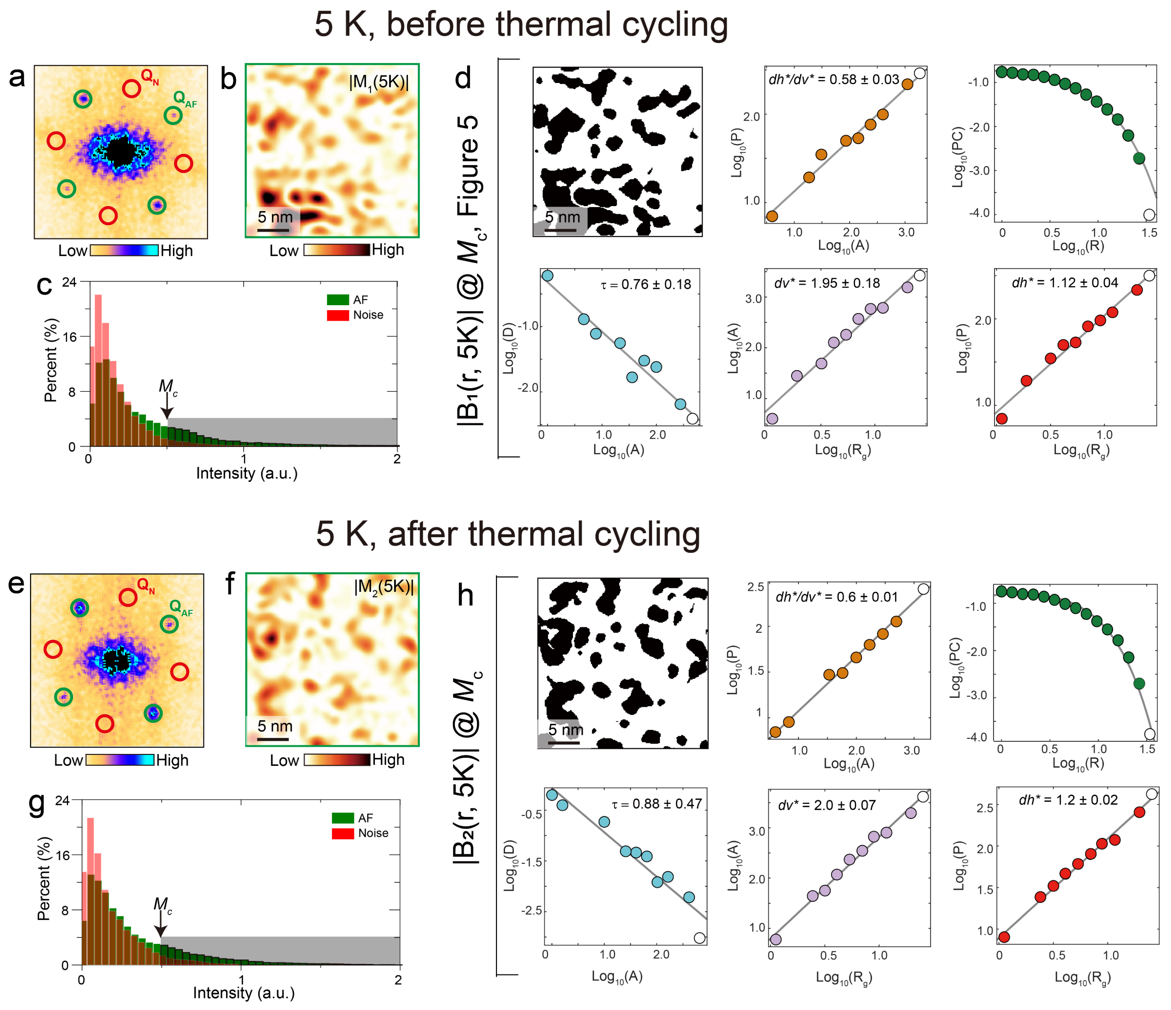

Figure S5. **Percolation analysis on $|M\,(\mathbf{r}, 5\ \mathrm{K})|$ maps before/after ther-mal cycling.** (a) The Fourier transform (FT) of the $M_1(\mathbf{r}, 5K)$ map (Figure S8b). The green circles cover the antiferromagnetic modulation wavevectors Q_{AF} , based on which we acquire the (b) magnetic amplitude $|M_1(\mathbf{r}, 5\ \mathrm{K})|$ map. Those red circles (same radius as the green ones, but rotated by 45 degrees) are used to create the rep-resentative noise amplitude map. (c) Histogram of the map in (b) (green) and noise amplitude map (partially transparent red). M_c denotes the threshold value to create the binarized AF domain map M_{c} in (d) (Figure S5). The dark clusters represent the regions with magnetic modulation amplitude higher than the M_c, corresponding to the columns of histogram covered by the partially black rectangle. The intensities of 5% pixels of the noise map and 25% of (b) are above M_{c}. (d) The corresponding plots display the results of statistical analysis of the cluster geometric metrics. The gray lines and curves denote the power-law fittings to the data after excluding empty circles, which represent clusters either touching boundaries of the FOV or only en-compassing a single pixel. Calculated exponents are listed in each plot. Similarly to Figure 5, values of P , R, and R_g are in the units of pixels and A is in the units of area occupied by a single pixel. The single pixel size in each binary image is 1.2 Å by 1.2 Å. (e-h) The same analysis applied to the map taken at 5 K, but after the thermal cycling using the same threshold as in (c). The intensities of 5% pixels in the noise map and 21% of $|M_2(\mathbf{r}, 5\ \mathrm{K})|$ map are above M_{c}.

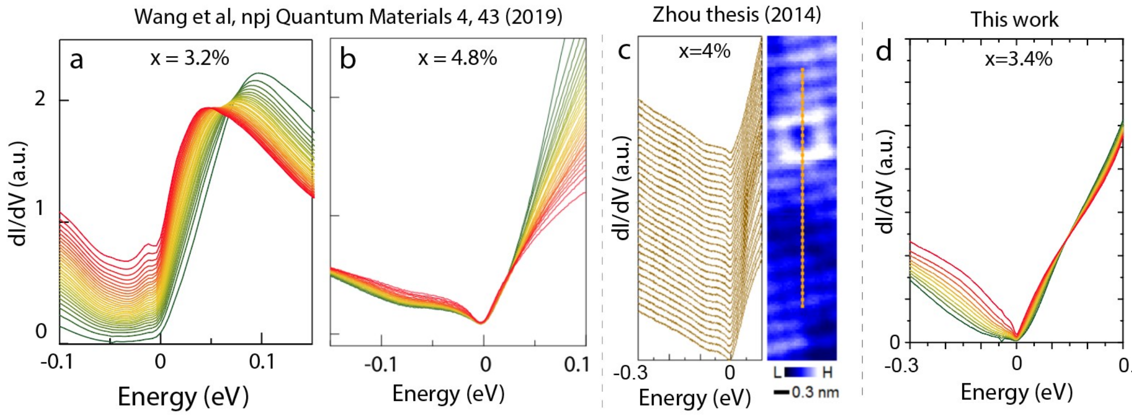

Figure S6. **Comparison of differential conductance dI/dV spectra between different experiments**. (a-b) Binned spectra for 3.2%, 4.8% (adapted from Ref. 27), (c) dI/dV linecut of 4% sample (adapted from Ref. 38), and (d) binned dI/dV spectra on 3.4% sample (this work). Differential conductance at -40 mV were chosen to classify the spectra in (a, b, d). STM setup condition: (c): V_{sample}= 300 mV, I_{set} = 300 pA, V_{exc} = 6 mV, 0 T

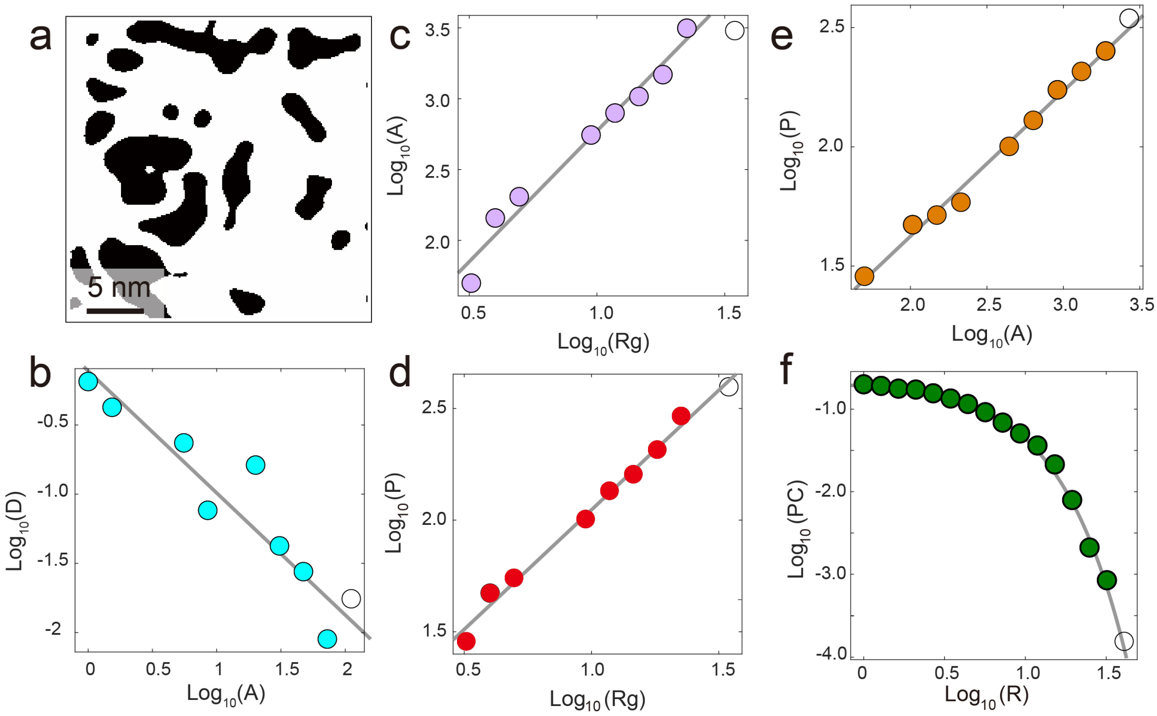

Figure S7. **Another data set demonstrating the scale-invariant magnetic texture in 3.4% La-doped Ir-327 at 5 K.** (a) Binarized AF domains obtained from $|M(\mathbf{r}, 5\text{ K})|$ map over another field-of-view that is several micrometers away from the one in Figure 5. The threshold M_c is determined by the same method that we used in Figure 5. (b) A plot of logarithmically binned domain area distribution, following a power-law distribution $D(A) \sim A^{-\tau}$, with $\tau = 0.86 \pm 0.25$. (c,d) Plots of Area (A) and Perimeter(P) vs. gyration radius (R_g) using logarithmic binning. Solid lines are power-law fits of $P \sim R_g^{dh^*}$ and $A \sim R_g^{dv^*}$ with critical exponents $dh^* = 1.1 \pm 0.04$ and $dv^* = 1.84 \pm 0.31$. (e) Perimeter(P) vs. Area (A) plot. The solid line is the power-law fit of $P \sim A^{dh^*/dv^*}$ with $dh^*/dv^* \sim 3/5$. (f) Pair connectivity (PC) function vs. distance (r) plot using logarithmic binning. The solid line is fit to a power-law function with an exponential cutoff $g_{conn} \sim r^{-\eta} \cdot e^{-x/\xi}$, where $\eta = -0.12 \pm 0.05$ is the exponent for the connectivity function. Values of P, r and R_g are in unit of pixels and A is in units of area occupied by a single pixel. The single pixel size in the raw STM topograph used to obtain image in (a) is 1.32 Å by 1.32 Å. STM setup condition: (a): $V_{\text{sample}} = 600$ mV, $I_{\text{set}} = 100$ pA, ± 2 T.

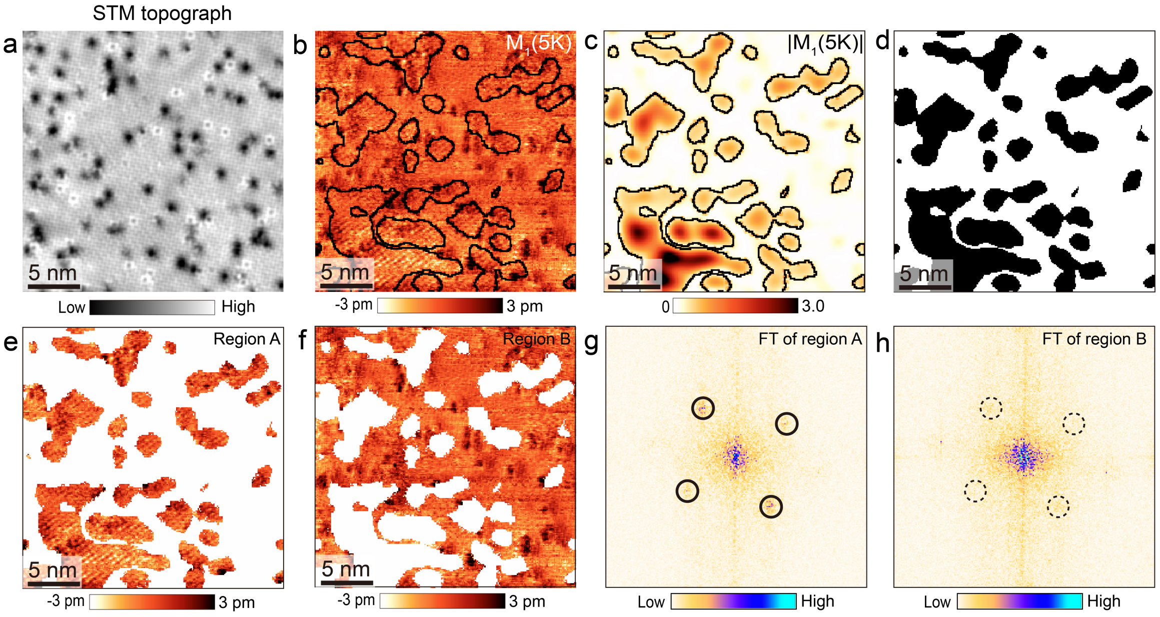

Figure S8. **Reliability proof of the magnetic domain analysis algorithm.** (a) STM topograph, (b) $M_1(r, 5K)$ map, (c) AF amplitude map $|M_1(\mathbf{r}, 5K)\ |$, and (d) corresponding binarized AF domain map (also in Figure 5a) over the same area of the $x \sim 0.034$ La-doped $Sr_3Ir_2O_7$ sample. AF domain outlines are obtained from (c) as described in the Supplementary Information 2 and superimposed in (b). (e) Regions in the $M(r)$ map with strong AF modulations, and (f) remaining regions not captured in (e). (g,h) Fourier transforms of (e,f), respectively.

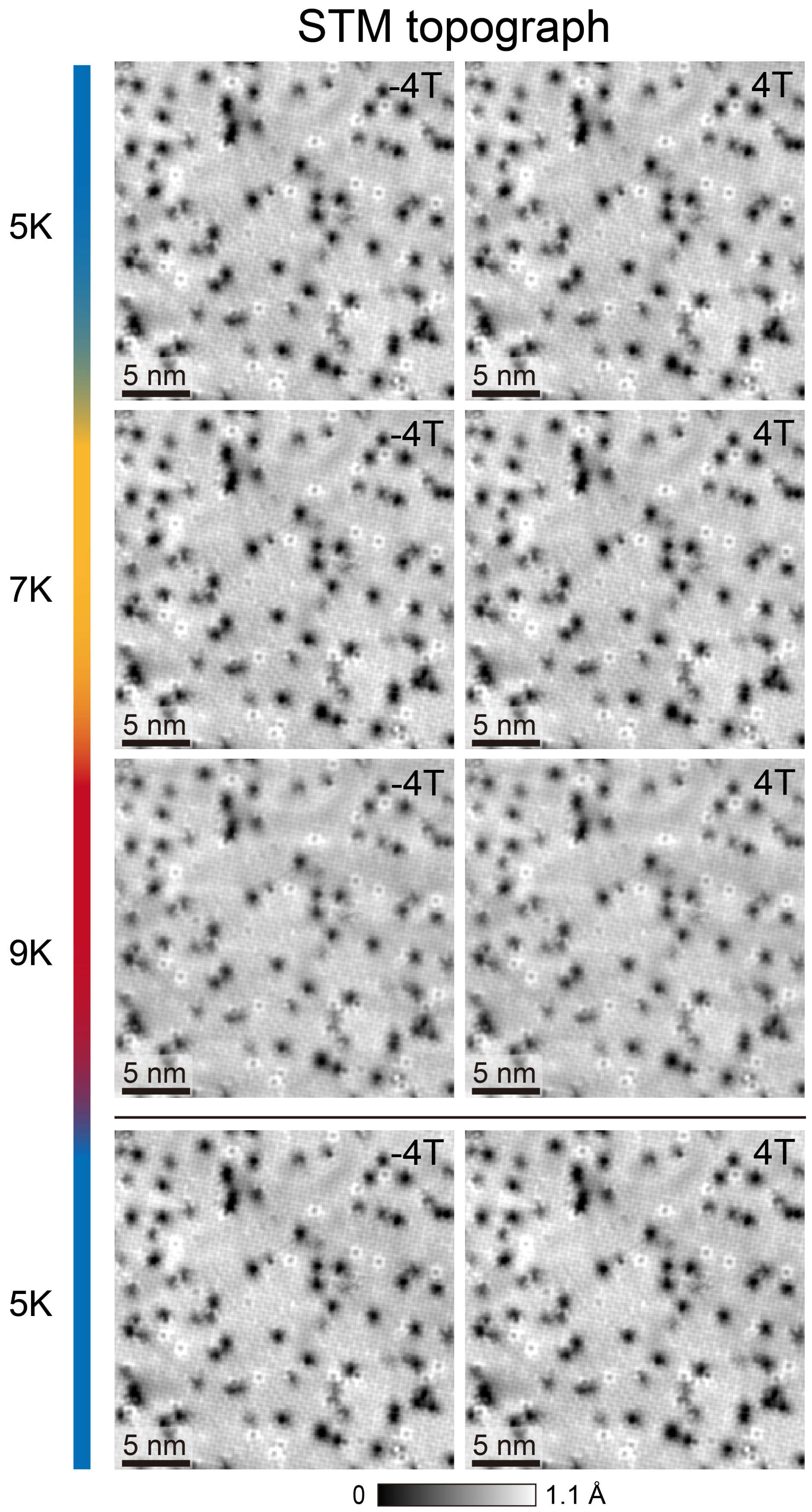

Figure S9. **STM topographs as a function of temperature.** The STM topographs taken at +/- 4 T as a function of temperature during a thermal cycle, acquired over the same area as Figure 3a. STM setup condition: V_{sample}= 600 mV, I_{set} = 100 pA, ±4 T.

REFERENCES AND NOTES

1. K. M. Lang, V. Madhavan, J. E. Hoffman, E. W. Hudson, H. Eisaki, S. Uchida, J. C. Davis, Imaging the granular structure of high-T_c superconductivity in underdoped $Bi_2Sr_2CaCu_2O_{8+\delta}$. *Nature* **415**, 412–416 (2002).

2. M. Uehara, S. Mori, C. H. Chen, S.-W. Cheong, Percolative phase separation underlies colossal magnetoresistance in mixed-valent manganites. *Nature* **399**, 560–563 (1999).

3. G. Campi, A. Bianconi, N. Poccia, G. Bianconi, L. Barba, G. Arrighetti, D. Innocenti, J. Karpinski, N. D. Zhigadlo, S. M. Kazakov, M. Burghammer, M. V. Zimmermann, M. Sprung, A. Rici, Inhomogeneity of charge-density-wave order and quenched disorder in a high-T_c superconductor. *Nature* **525**, 359–362 (2015).

4. Y. Okada, D. Walkup, H. Lin, C. Dhital, T.-R. Chang, S. Khadka, W. Zhou, H.-T. Jeng, M. Paranjape, A. Bansil, Z. Wang, S. D. Wilson, V. Madhavan, Imaging the evolution of metallic states in a correlated iridate. *Nat. Mater.* **12**, 707–713 (2013).

5. I. Battisti, K. M. Bastiaans, V. Fedoseev, A. de la Torre, N. Iliopoulos, A. Tamai, E. C. Hunter, R. S. Perry, J. Zaanen, F. Baumberger, M. P. Allan, Universality of pseudogap and emergent order in lightly doped Mott insulators. *Nat. Phys.* **13**, 21–25 (2017).

6. C. Kittel, Physical theory of ferromagnetic domains. *Rev. Mod. Phys.* **21**, 541–583 (1949).

7. L. Zhang, C. Israel, A. Biswas, R. L. Greene, A. de Lozanne, Direct observation of percolation in a manganite thin film. *Science* **298**, 805–807 (2002).

8. A. Fert, N. Reyren, V. Cros, Magnetic skyrmions: Advances in physics and potential applications. *Nat. Rev. Mater.* **2**, 17031 (2017).

9. N. Romming, C. Hanneken, M. Menzel, J. E. Bickel, B. Wolter, K. von Bergmann, A. Kubetzka, R. Wiesendanger, Writing and deleting single magnetic skyrmions. *Science* **341**, 636–639 (2013).

10. H. Jani, J.-C. Lin, J. Chen, J. Harrison, F. Maccherozzi, J. Schad, S. Prakash, C.-B. Eom, A. Ariando, T. Venkatesan, P. G. Radaelli, Antiferromagnetic half-skyrmions and bimerons at room temperature. *Nature* **590**, 74–79 (2021).

11. J. Li, J. Pelliciari, C. Mazzoli, S. Catalano, F. Simmons, J. T. Sadowski, A. Levitan, M. Gibert, E. Carlson, J.-M. Triscone, S. Wilkins, R. Comin, Scale-invariant magnetic textures in the strongly correlated oxide $NdNiO_3$. *Nat. Commun.* **10**, 4568 (2019).(2019)

12. B. Kim, H. Jin, S. Moon, J.-Y. Kim, B.-G. Park, C. Leem, J. Yu, T. Noh, C. Kim, S.-J. Oh, J.-H. Park, V. Durairaj, G. Cao, E. Rotenberg, Novel J_{eff}=1/2 mott state induced by relativistic spin-orbit coupling in Sr_2IrO_4. *Phys. Rev. Lett.* **101**, 076402 (2008).

13. J. G. Rau, E. K.-H. Lee, H.-Y. Kee, Spin-orbit physics giving rise to novel phases in correlated systems: Iridates and related materials. *Annu. Rev. Condens. Matter Phys.* **7**, 195–221 (2016).

14. G. Cao, P. Schlottmann, The challenge of spin-orbit-tuned ground states in iridates: A key issues review. *Rep. Prog. Phys.* **81**, 042502–042558 (2018).

15. J. Bertinshaw, Y. K. Kim, G. Khaliullin, B. J. Kim, Square lattice iridates. *Annu. Rev. Condens. Matter Phys.* **10**, 315–336 (2019).

16. H. Zhao, S. Manna, Z. Porter, X. Chen, A. Uzdejczyk, J. Moodera, Z. Wang, S. D. Wilson, I. Zeljkovic, Atomic-scale fragmentation and collapse of antiferromagnetic order in a doped Mott insulator. *Nat. Phys.* **15**, 1267–1272 (2019).

17. G. Mattoni, P. Zubko, F. Maccherozzi, A. J. H. van der Torren, D. B. Boltje, M. Hadjimichael, N. Manca, S. Catalano, M. Gibert, Y. Liu, J. Aarts, J.-M. Triscone, S. S. Dhesi, A. D. Caviglia, Striped

nanoscale phase separation at the metal–insulator transition of heteroepitaxial nickelates. *Nat. Commun.* **7**, 13141 (2016).

18. A. S. McLeod, E. van Heumen, J. G. Ramirez, S. Wang, T. Saerbeck, S. Guenon, M. Goldflam, L. Anderegg, P. Kelly, A. Mueller, M. K. Liu, I. K. Schuller, D. N. Basov, Nanotextured phase coexistence in the correlated insulator V_2O_3. *Nat. Phys.* **13**, 80–86 (2017).

19. K. McElroy, J. Lee, J. A. Slezak, D.-H. Lee, H. Eisaki, S. Uchida, J. C. Davis, Atomic-scale sources and mechanism of nanoscale electronic disorder in $Bi_2Sr_2CaCu_2O_{8+\delta}$. *Science* **309**, 1048–1052 (2005).

20. I. Zeljkovic, Z. Xu, J. Wen, G. Gu, R. S. Markiewicz, J. E. Hoffman, Imaging the impact of single oxygen atoms on superconducting $Bi_{2+y}Sr_{2-y}CaCu_2O_{8+x}$. *Science* **337**, 320–323 (2012).

21. I. Nagai, Y. Yoshida, S. I. Ikeda, H. Matsuhata, H. Kito, M. Kosaka, Canted antiferromagnetic ground state in $Sr_3Ir_2O_7$. *J. Phys. Condens. Matter* **19**, 136214 (2007).

22. C. Dhital, S. Khadka, Z. Yamani, C. de la Cruz, T. C. Hogan, S. M. Disseler, M. Pokharel, K. C. Lukas, W. Tian, C. P. Opeil, Z. Wang, S. D. Wilson, Spin ordering and electronic texture in the bilayer iridate $Sr_3Ir_2O_7$. *Phys. Rev. B* **86**, 100401 (2012).

23. C. Dhital, T. Hogan, W. Zhou, X. Chen, Z. Ren, M. Pokharel, Y. Okada, M. Heine, W. Tian, Z. Yamani, C. Opeil, J. S. Helton, J. W. Lynn, Z. Wang, V. Madhavan, S. D. Wilson, Carrier localization and electronic phase separation in a doped spin-orbit-driven Mott phase in $Sr_3(Ir_{1-x}Ru_x)_2O_7$. *Nat. Commun.* **5**, 3377 (2014).

24. T. Hogan, Z. Yamani, D. Walkup, X. Chen, R. Dally, T. Z. Ward, M. P. M. Dean, J. Hill, Z. Islam, V. Madhavan, S. D. Wilson, First-order melting of a weak spin-orbit mott insulator into a correlated metal. *Phys. Rev. Lett.* **114**, 257203 (2015).

25. R. Wiesendanger, Spin mapping at the nanoscale and atomic scale. *Rev. Mod. Phys.* **81**, 1495–1550 (2009).

26. X. Lu, D. E. E. McNally, M. Moretti Sala, J. Terzic, M. H. H. Upton, D. Casa, G. Ingold, G. Cao, T. Schmitt, Doping evolution of magnetic order and magnetic excitations in $(Sr_{1-x}La_x)_3Ir_2O_7$. *Phys. Rev. Lett.* **118**, 027202 (2017).

27. Z. Wang, D. Walkup, Y. Maximenko, W. Zhou, T. Hogan, Z. Wang, S. D. Wilson, V. Madhavan, Doping induced Mott collapse and possible density wave instabilities in $(Sr_{1-x}La_x)_3Ir_2O_7$. *npj Quantum Mater.* **4**, 43 (2019).

28. B. Phillabaum, E. W. Carlson, K. A. Dahmen, Spatial complexity due to bulk electronic nematicity in a superconducting underdoped cuprate. *Nat. Commun.* **3**, 915 (2012).

29. S. Liu, B. Phillabaum, E. W. Carlson, K. A. Dahmen, N. S. Vidhyadhiraja, M. M. Qazilbash, D. N. Basov, Random field driven spatial complexity at the mott transition in VO_2. *Phys. Rev. Lett.* **116**, 036401 (2016).

30. W. Post, A. S. McLeod, M. Hepting, M. Bluschke, Y. Wang, G. Cristiani, G. Logvenov, A. Charnukha, G. X. Ni, P. Radhakrishnan, M. Minola, A. Pasupathy, V. A. Boris, E. Benckiser, K. A. Dahmen, E. W. Carlson, B. Keimer, D. N. Basov, Coexisting first- and second-order electronic phase transitions in a correlated oxide. *Nat. Phys.* **14**, 1056–1061 (2018).

31. M. Newman, Power laws, Pareto distributions and Zipf's law. *Contemp. Phys.* **46**, 323–351 (2005).

32. D. Stauffer, A. Aharony, *Introduction to Percolation Theory* (Taylor & Francis, 2018).

33. C. Jin, Z. Tao, K. Kang, K. Watanabe, T. Taniguchi, K. F. Mak, J. Shan, Imaging and control of critical fluctuations in two-dimensional magnets. *Nat. Mater.* **19**, 1290–1294 (2020).

34. M. J. Lawler, K. Fujita, J. Lee, A. R. Schmidt, Y. Kohsaka, C. K. Kim, H. Eisaki, S. Uchida, J. C. Davis, J. P. Sethna, E.-A. Kim, Intra-unit-cell electronic nematicity of the high-T_c copper-oxide pseudogap states. *Nature* **466**, 347–351 (2010).

35. W. Janke, A. M. J. Schakel, Fractal structure of spin clusters and domain walls in the two-dimensional Ising model. *Phys. Rev. E* **71**, 036703 (2005).

36. R. M. Feenstra, Tunneling spectroscopy of the (110) surface of direct-gap III-V semiconductors. *Phys. Rev. B* **50**, 4561–4570 (1994).

37. W. Ruan, X. Li, C. Hu, Z. Hao, H. Li, P. Cai, X. Zhou, D. Lee, Y. Wang, Visualization of the periodic modulation of Cooper pairing in a cuprate superconductor. *Nat. Phys.* **14**, 1178–1182 (2018).

38. W. Zhou, "STM probe on the surface electronic states of spin-orbit coupled materials," thesis, Boston College, Chestnut Hill, MA (2014).
